# Low expression of PRRG2 in kidney renal clear cell carcinoma: an immune infiltration-associated prognostic biomarker

**DOI:** 10.1007/s12672-024-00864-x

**Published:** 2024-01-16

**Authors:** Gonglin Tang, Guixin Ding, Gang Wu, Xiaofeng Wang, Tianqi Wang, Qingsong Zou, Kai Sun, Jitao Wu

**Affiliations:** 1 Department of Urology, The Affiliated Yantai Yuhuangding Hospital of Qingdao University, No. 20 East Yuhuangding Road, Yantai, 264000 Shandong China; 2 Urology Department, Shandong Provincial Hospital, Shandong University, Jinan, 250021 China

**Keywords:** Kidney renal clear cell carcinoma, PRRG2, Prognostic biomarker, Immune infiltration

## Abstract

**Objective:**

This study aims to explore the prognostic significance of Proline-rich γ-carboxyglutamic acid protein 2 (PRRG2) in Kidney Renal Clear Cell Carcinoma (KIRC), a prevalent and deadly cancer, and its association with immune cell infiltration, a key strategy in developing effective biomarkers.

**Methods:**

The study meticulously elucidated the prognostic significance and potential role of PRRG2 in KIRC, correlating its expression with patient sex, age, metastasis, and pathological stage. Utilizing Gene Ontology (GO), Kyoto Encyclopedia of Genes and Genomes (KEGG), and Gene Set Enrichment Analysis (GSEA), the involvement of PRRG2 in immune response was investigated. The association between PRRG2 expression and immune cell infiltration was also scrutinized. Ultimately, cellular and tissue identity were confirmed via immunohistochemical staining and quantitative real-time PCR.

**Results:**

The study elucidates a notable decrease in PRRG2 expression in KIRC patients, correlating with demographic factors, metastasis, and pathological staging, and portending an unfavorable prognosis. Bioinformatic analyses underscore PRRG2’s role in immune response, with its expression significantly tied to immune cell infiltration and marker expression.

**Conclusion:**

PRRG2 may potentially impact prognosis in KIRC patients by regulating immune infiltration, thus rendering PRRG2 a promising candidate prognostic biomarker for KIRC-associated immune infiltration.

**Supplementary Information:**

The online version contains supplementary material available at 10.1007/s12672-024-00864-x.

## Introduction

Renal Cell Carcinoma (RCC) represents a prevalent and deadly neoplasm within the global genitourinary system. RCC encompasses a variety of subtypes, such as Clear Cell Renal Cell Carcinoma (KIRC), Chromophobe Renal Cell Carcinoma, Papillary Renal Cell Carcinoma, and Collecting Duct Carcinoma. KIRC, colloquially referred to as Clear Cell Renal Cell Carcinoma, constitutes over 80% of all RCC instances [1]. Among various cancers, KIRC is known for its strong immune infiltration [2]. Recently, immune checkpoint inhibitors (ICIs) have been shown to be highly effective against this disease [3]. The tumor microenvironment (TME) plays a crucial role in KIRC cell biology, affecting both prognosis and treatment response [2]. Moreover, there are few molecular biomarkers and therapeutic targets available for KIRC in clinical diagnosis and prognosis evaluation [4]. Hence, it is of paramount importance to decipher the diverse tumor microenvironment of KIRC, unearth novel prognostic markers and therapeutic objectives, and amalgamate them with immune infiltration.

Over a decade ago, the identification and characterization of the genes that encode a family of proteins known as proline-rich γ-carboxyglutamic acid (Gla) (PRRG) proteins transpired. However, their functions remain enigmatic [5]. Proline-rich Gla protein 2 (PRRG2) represents one of the four known transmembrane-carboxyglutamic acid proteins in vertebrates. As a founding member of the vitamin K-dependent, single-pass integral membrane protein family, PRRG2 is characterized by an extracellular N-terminus consisting of approximately 45 amino acids rich in Gla, as well as a conserved Pro/Leu-Pro-xaa-Tyr (PY) motif proximal to its C-terminus [6, 7]. PRRG2 primarily localizes to the cell surface, where the Gla domain is exposed extracellularly, serving as a pivotal gene node with positive autoregulatory function that plays a critical role in maintaining human hemostasis [8, 9]. Despite the restricted expression of Gla protein in hepatic tissue within the coagulation cascade system, PRRG protein is expressed in several extrahepatic sites [6, 7]. In the cytoplasmic domain, PRRG2 may possess SH3 and WW binding motifs [6], whose interactions regulated by WW domains are implicated in several diseases, including cancer and neurological disorders [10–13]. Additionally, PRRG2 has been shown to interact with two proteins, YAP1 and NEDD4 [7, 14]. The latter is a ubiquitin ligase, while the former is a downstream target of the hippo tumor suppressor pathway [15]. Albeit, hitherto, inquiries pertaining to PRRG2 in KIRC remain unexplored, rendering its mechanism of action enigmatic.

The objective of this investigation is to amalgamate diverse bioinformatics approaches to scrutinize the correlation between PRRG2 and KIRC metastasis and immune infiltration, and to delve into its molecular regulation. Our discoveries unveil a substantial diminution of PRRG2 expression in KIRC tissue relative to non-neoplastic tissue. Furthermore, the diminished expression of PRRG2 is inversely associated with the prognosis of KIRC patients. In addition, the expression of PRRG2 in KIRC tissue is intimately linked with the infiltration of various immune cells, potentially influencing prognosis.

## Materials and methods

### Database and bioinformatics analysis

We used Oncomine (www.oncomine.org), a microarray database, to examine gene expression levels of PRRG2 in KIRC tissues. The gene rank was set to all, the foldchange to 1.5, and the *p*-value to 0.05.

As we delved into the realm of KIRC tissues, the expression of PRRG2 via UALCAN (http://ualcan.path.uab.edu/) was scrutinized. In doing so, we sought to uncover its correlation with various clinicopathological parameters, including but not limited to gender, tumor stage, lymph node metastasis, and age.

GEPIA (http://gepia.cancer-pku.cn/index.html) serves as a gateway for gene expression analysis, utilizing the TCGA and GTEX databases. In the present investigation, we scrutinized the expression analysis of PRRG2 by means of the TCGA-KIRC dataset. Leveraging the "expression DIY" module of GEPIA, along with TCGA normal and GTEX data matching, a logarithmic scale of log2 (TPM + 1) was employed to assess the expression of PRRG2 in kidney tissue samples from both KIRC and normal individuals.

We have chosen two KIRC datasets comprising 1426 cases for further analysis, utilizing the cBioPortal. Our study entails a comprehensive evaluation of the type and frequency of genomic aberrations in PRRG2 within KIRC tissues, along with a thorough assessment of the overall survival (OS) and disease-free survival (DFS) of PRRG2.

The investigation of PRRG2's biological function in KIRC entails an exploration of the Gene Ontology (GO) and Kyoto Encyclopedia of Genes and Genomes (KEGG) databases. To uncover PRRG2's potential mode of action, Gene Set Enrichment Analysis (GSEA) is employed. The R package ClusterProfiler is utilized to conduct GO, KEGG, and GSEA analyses.

We employed the Tumor Immune Estimation Resource (TIMER) to assess the expression of PRRG2 across a diverse range of malignancies, while simultaneously analyzing the infiltration of various immune cell populations for a comprehensive appraisal. In addition, we leveraged the TCGA database to probe the correlation between PRRG2 expression and immune cell infiltration, specifically B cells, CD8 + T cells, CD4 + T cells, neutrophils, macrophages, and dendritic cells. Moreover, we utilized TIMER to investigate the connection between PRRG2 expression and multiple gene marker sets of immune cells, as well as to evaluate the association between PRRG2 expression and immunological infiltration.

Our research evaluated the proportion of immune cells that penetrated the tumor in KIRC through application of CiberSort (https://cibersort.stanford.edu/). Additionally, we examined the correlation between prgg2 expression and subsets of immune cells. To determine the possible influence of PRRG2 expression on lymphocytes, we established a threshold of a *p*-value less than 0.05.

The KM Plotter (http://kmplot.com) is an online repository of gene expression data and survival records for KIRC patients. We employed this resource to scrutinize the prognostic significance of PRRG2 in KIRC. Patient specimens were categorized into high and low expression groups using the median expression value, and the HR, CI, and log *p*-values for overall survival (OS), disease-specific survival (DSS), and progression-free interval (PFI) were evaluated for each group.

Utilizing the PrognoScan repository (http://www.abren.net/PrognoScan/), we scrutinized the PRRG2 expression in KIRC tissues in relation to survival metrics, such as OS and relapse-free survival (RFS). A computed HR with 95% CIs was employed, and the cutoff was adjusted to a Cox *p*-value of less than 0.05.

We established an interactive PRRG2 network through the utilization of the GeneMANIA database (http://www.genemania.org). Furthermore, an assessment of the PRRG2 protein–protein interaction (PPI) network was carried out by examining the STRING online database (https://string-db.org/).

### Patient samples

During the period from 2020 to 2023, a total of 40 patients who underwent radical or partial nephrectomy in the Department of Urology at Yantai Yuhuangding Hospital affiliated with Qingdao University provided us with KIRC tissue and matched normal kidney tissue. The surgical specimens were immediately immersed in liquid nitrogen and subsequently transferred to a − 80 °C freezer for further use. All participants signed informed consent forms, and the project was approved by the Ethics Committee of Yantai Yuhuangding Hospital affiliated with Qingdao University.

### Cell lines and cell culture

The human normal renal proximal convoluted tubular cell line (HK-2) and clear cell renal cell carcinoma (ccRCC) cell lines (ACHN, 769-P, Caki-2, 786-O) were procured from the Cell Bank of the Chinese Academy of Sciences. DMEM (BI, Israel) was utilized for the cultivation of HK-2 cells, while RPMI1640 (BI, Israel) was used for the other cells. All media were supplemented with 10% fetal bovine serum (FBS) and 1% penicillin and streptomycin. The cells were cultured in a humidified incubator at 37 °C with 5% CO_2_.

### RNA extraction, reverse transcription and quantitative real‑time PCR (qRT-PCR)

We utilized the SteadyPure Rapid RNA Extraction Kit (Accurate Biosciences, China) to extract total RNA from freshly frozen tissues or KIRC cell lines, followed by reverse transcription using the Evo M-MLV RT Mix Kit (Accurate Biology, China). Subsequently, we performed qPCR using the SYBR^®^ Green Premix Pro Taq HS qPCR Kit and Rox Reference Dye (Accurate Biology, China). The internal reference gene, GAPDH, was employed, and the relative expression level of the target gene was estimated using the 2^−ΔΔCT^ computational technique. Each experiment was conducted in triplicate, and the primer sequences used are presented in Table 1.

**Table 1:** Sequence of gene-specific primers for qPCR

Gene	Forward sequence (5′–3′)	Reverse sequence (5′–3′)
PRRG2	GCGCTTTTGGGAGAGCTACATC	CAGCGCAGATACCAAAAGGCTC
GAPDH	GTCTCCTCTGACTTCAACAGCG	ACCACCCTGTTGCTGTAGCCAA

### Immunohistochemical (IHC) analysis

Immunohistochemical staining was conducted utilizing a Roche Bench Mark GX semi-automated immunohistochemistry system for identification of cancerous and corresponding para-cancerous tissues. Subsequently, the slender incision was stained with hematoxylin and eosin to generate a more elaborate visualization of the tissues. Under high magnification, ten fields of view are randomly selected, with scoring based on the intensity of positive staining: cells exhibiting a light yellow hue are assigned a score of 1; cells stained yellow receive a score of 2, while those stained brown are given a score of 3. The percentage of positively stained cells is evaluated as follows: a score of 0 corresponds to less than 10%; a score of 1 corresponds to 11–25%; a score of 2 corresponds to 26–50%; a score of 3 corresponds to 51–75%; a score of 4 corresponds to over 75%. The immunohistochemical score is determined by multiplying the two scores.

### Statistical analysis

Oncomine produces results that comprise of *p*-values, fold changes, and rankings. The outcomes of Kaplan–Meier plots, PrognoScan, and GEPIA are displayed with HR and P or Cox *p*-values from a log-rank test. The heat map illustrating the associations between PRRG2 associated genes was generated using the R software program. Spearman's correlation and statistical significance were employed to evaluate the correlation of gene expression. The experimental data were analyzed using two-sample *t*-tests or analysis of variance (ANOVA) for more than two groups in SPSS 23.0. Statistical significance was determined for *p*-values less than 0.05.

## Results

### PRRG2 expression is reduced in KIRC patients

**Fig. 1:**
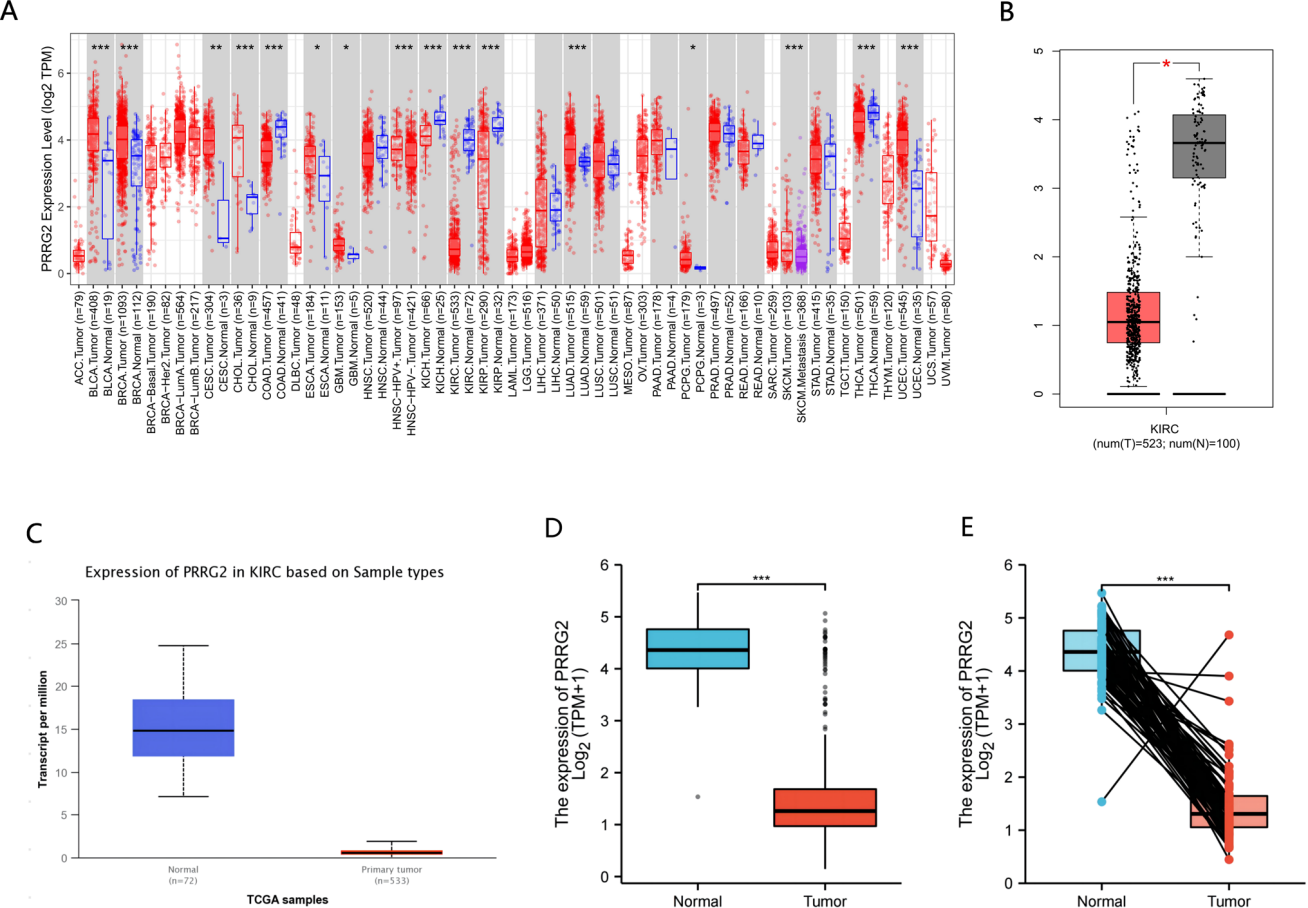
**A** The TIMER database was utilized to investigate the expression of PRRG2 in various cancer types. **B** The GEPIA database revealed altered PRRG2 expression in KIRC compared to normal tissue. **C** Examination of PRRG2 expression in KIRC was conducted using the UALCAN database. **D** Analysis of PRRG2 expression in KIRC and adjacent normal tissues was performed using the TCGA database. **E** PRRG2 expression in 52 pairs of KIRC tissues and adjacent normal tissues was statistically analyzed using the TCGA database. *p < 0.05, **p < 0.01, ***p < 0.001

The TIMER online database was utilized to initially scrutinize PRRG2 mRNA manifestation in human malignancies. We scrutinized PRRG2 expression in 36 carcinomas and observed a significant decrease in expression in KIRC as compared to normal tissues (Fig. 1a). Additionally, we unearthed that PRRG2 expression was lower in KIRC tissues than in normal kidney tissues in the GEPIA and UALCAN databases (Fig. 1b, c). Moreover, by utilizing data directly provided from TCGA, we probed the expression of PRRG2 in KIRC samples as well as neighboring normal tissues. PRRG2 expression was shown to be considerably higher in KIRC tissues (Fig. 1d). Furthermore, it was ascertained that the expression of PRRG2 in 56 KIRC patients was significantly lower than in adjacent normal samples (Fig. 1e). Based on these findings, we conclude that PRRG2 expression is downregulated in KIRC, thereby indicating that PRRG2 could play a significant regulatory role in the development of KIRC.

**Fig. 2:**
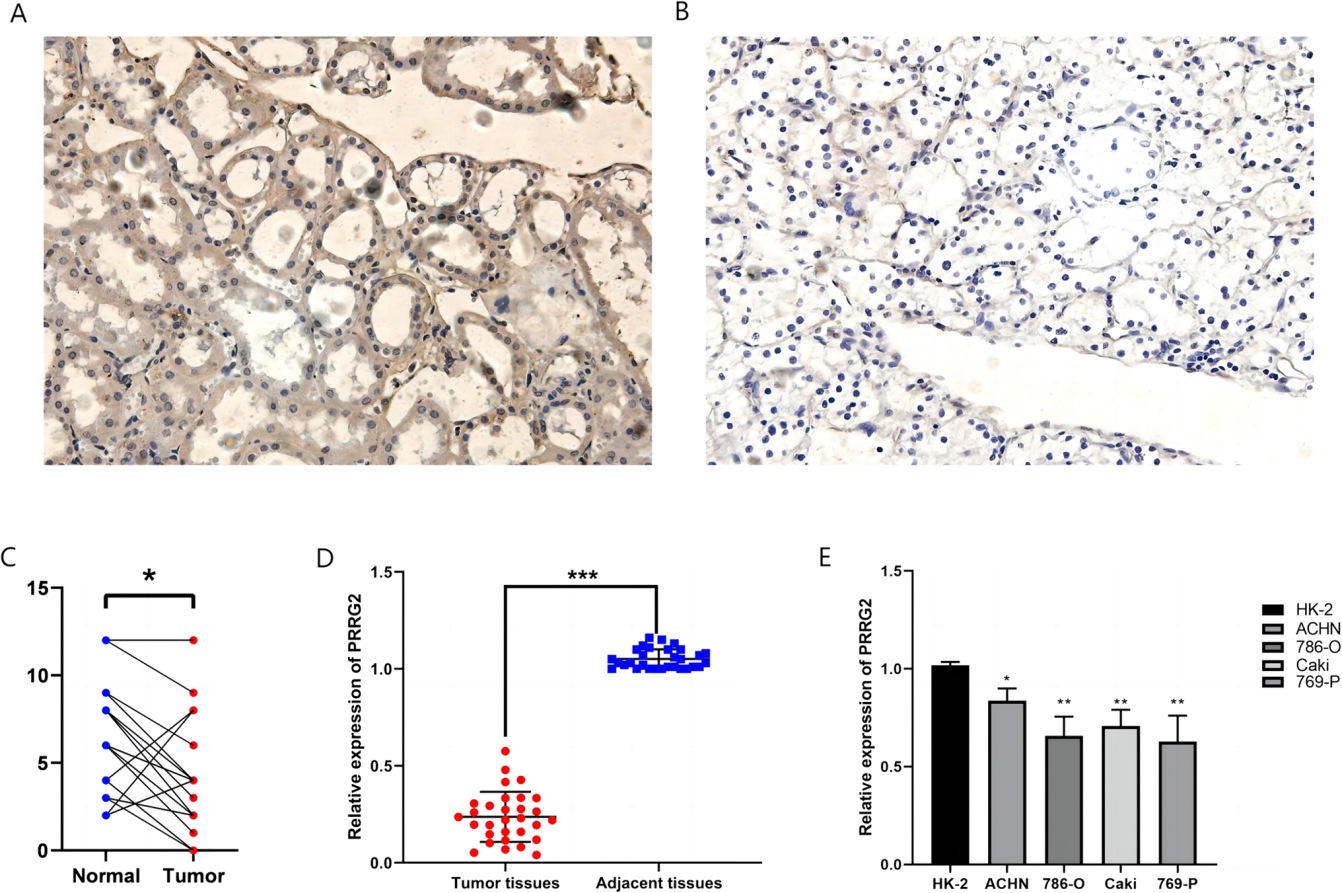
Protein and mRNA expression of PRRG2 in KIRC patients. **A** High expression of PRRG2 protein (× 200) in para-cancer tissue; **B** Low expression of PRRG2 protein (× 200) in cancer tissue. **C** Comparative analysis of PRRG2 expression in cancer and para-cancer tissue. **D** The mRNA expression levels of PRRG2 in KIRC tissues (T = 35, N = 35). **E** The mRNA expression levels of PRRG2 in KIRC cell lines. *p < 0.05, **p < 0.01, ***p < 0.001

We further scrutinized the protein expression of PRRG2 in KIRC tissues by utilizing immunohistochemistry and found that the protein level of PRRG2 in KIRC tissues was significantly lower than that in normal kidney tissues (Fig. 2a–c). We procured 32 pairs of tumor samples and surrounding tissues from KIRC patients for qRT-PCR verification. The results evinced that tumor tissues had substantially reduced levels of PRRG2 mRNA expression (Fig. 2d). This revelation was further corroborated in cell lines from kidney carcinoma, where the majority of kidney carcinoma cell lines exhibited significantly reduced PRRG2 mRNA levels as compared to HK-2 (Fig. 2e).

### PRRG2 expression and clinical parameters of KIRC

**Fig. 3:**
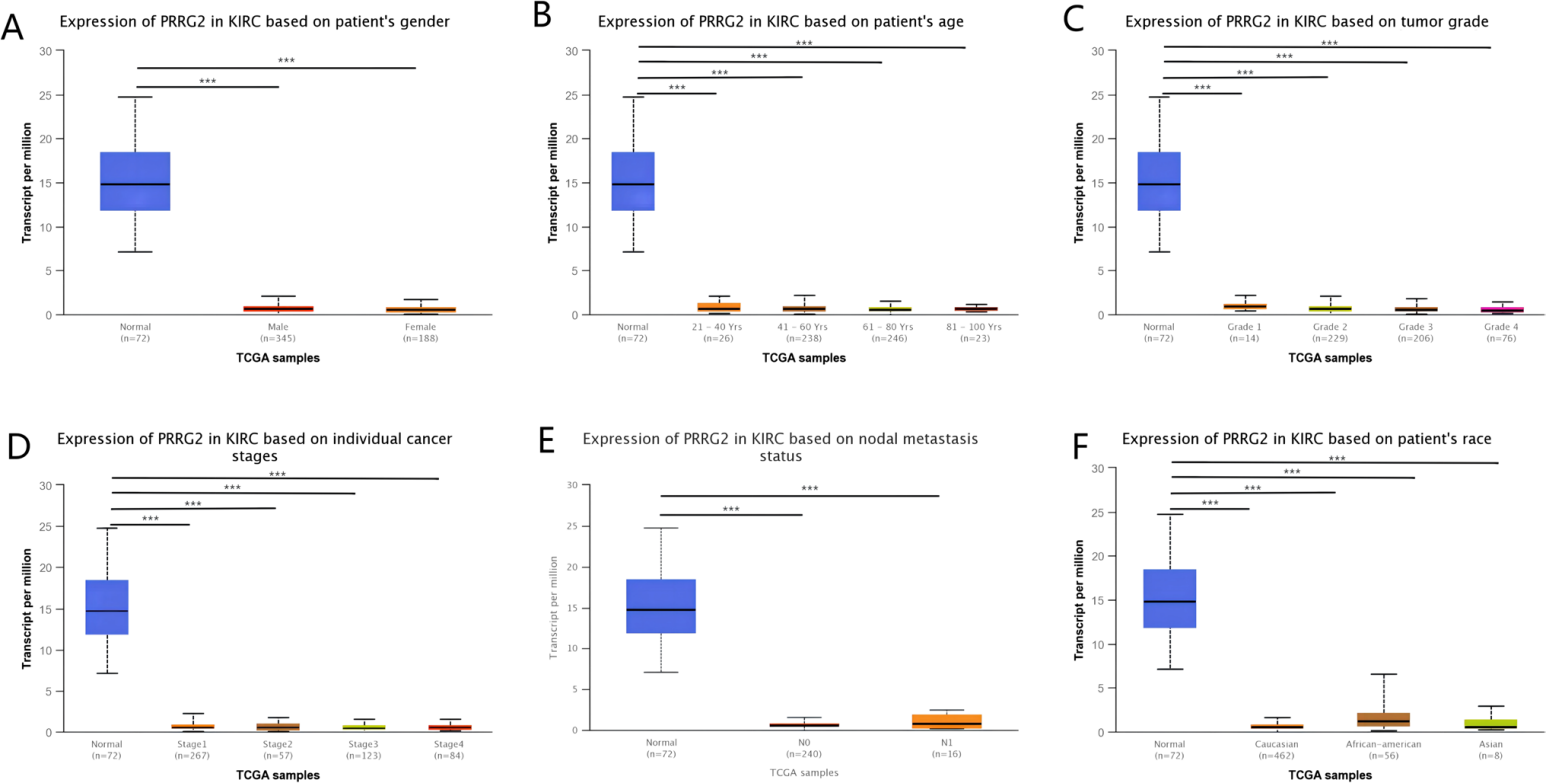
Box plots evaluating hepcidin expression among different groups of patients based on clinical parameters using the UALCAN database. Analysis is shown for sex (**A**), age (**B**), tumor grade (**C**), individual cancer stages (**D**), nodal metastasis status (**E**), and race (**F**). ***p < 0.001

We assessed PRRG2 expression in diverse patient groups based on clinical factors utilizing the UALCAN online tool. PRRG2 expression was markedly diminished in both male and female KIRC samples as compared to their normal counterparts (Fig. 3a). Furthermore, a significantly lower expression of PRRG2 was observed in KIRC patients in relation to age, tumor stage grade, and patient ethnicity (Fig. 3b–f). These findings suggest that PRRG2 expression is intricately linked to tumor growth and metastasis.

### Reduced PRRG2 expression is associated poor prognosis in KIRC patients

**Fig. 4:**
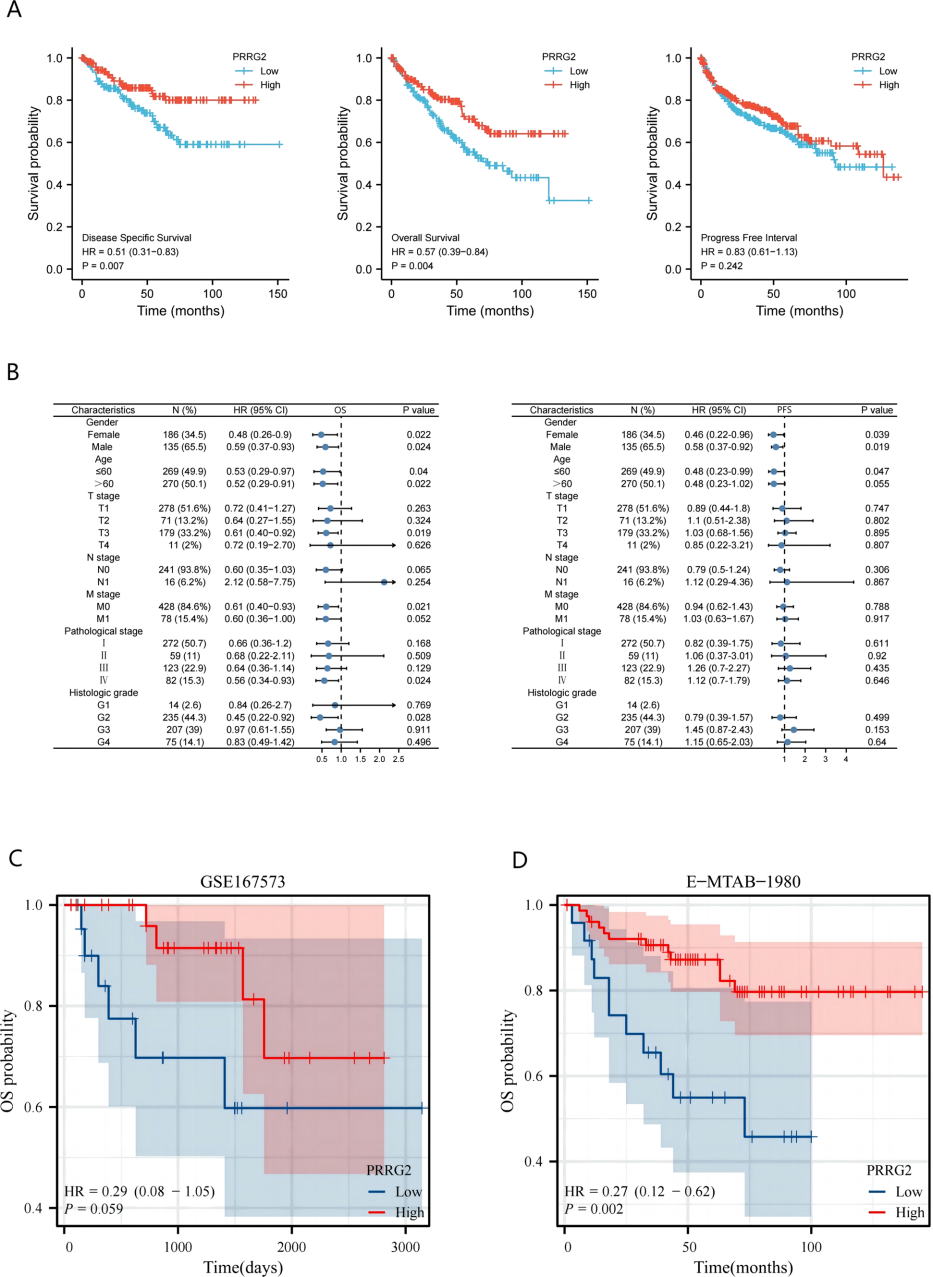
Survival curve evaluating the prognostic value of PRRG2. **A** Survival curves using the Kaplan–Meier plotter are shown for OS, PFI and DSS. **B** Forest plots show the correlation between PRRG2 expression and clinicopathological parameters in KIRC patients. **C**, **D** Kaplan–Meier analysis of OS in GEO and E-MTAB-1980 database

As PRRG2 expression is intricately linked to KIRC progression and metastasis, we delved into the prognostic value of the PRRG2 gene. According to the Kaplan Meier plotter, KIRC patients in the low expression group exhibited poorer overall survival (OS) and disease-specific survival (DSS), albeit no significant difference in the progression-free interval (PFI) (Fig. 4a, c, d). Our discoveries suggest a direct correlation between PRRG2 expression and the prognosis of KIRC patients.

### Validation of PRRG2's predictive value based on distinct clinicopathological characteristics

We explored the correlation between the PRRG2 gene expression in KIRC tissues and clinical features, utilizing the Kaplan Meier database, in order to gain deeper insights into the prognostic value and potential mechanisms of PRRG2 gene expression in KIRC. Our analysis revealed that low PRRG2 expression was significantly associated with poor OS, DSS and PFI in both male and female KIRC patients (Fig. 4a). Additionally, low PRRG2 expression was linked to OS in KIRC patients of all age ranges, those in T3 stage, m0 stage, pathological stage IV, and histological grade G2 (Fig. 4b). However, only KIRC patients aged ≤ 60 years showed a correlation with low PRRG2 expression in terms of PFS (Fig. 4b). These findings underscore the prognostic significance of PRRG2 mRNA expression in KIRC tissue.

### Identification of PRRG2 interacting genes and proteins and their genetic variations

**Fig. 5:**
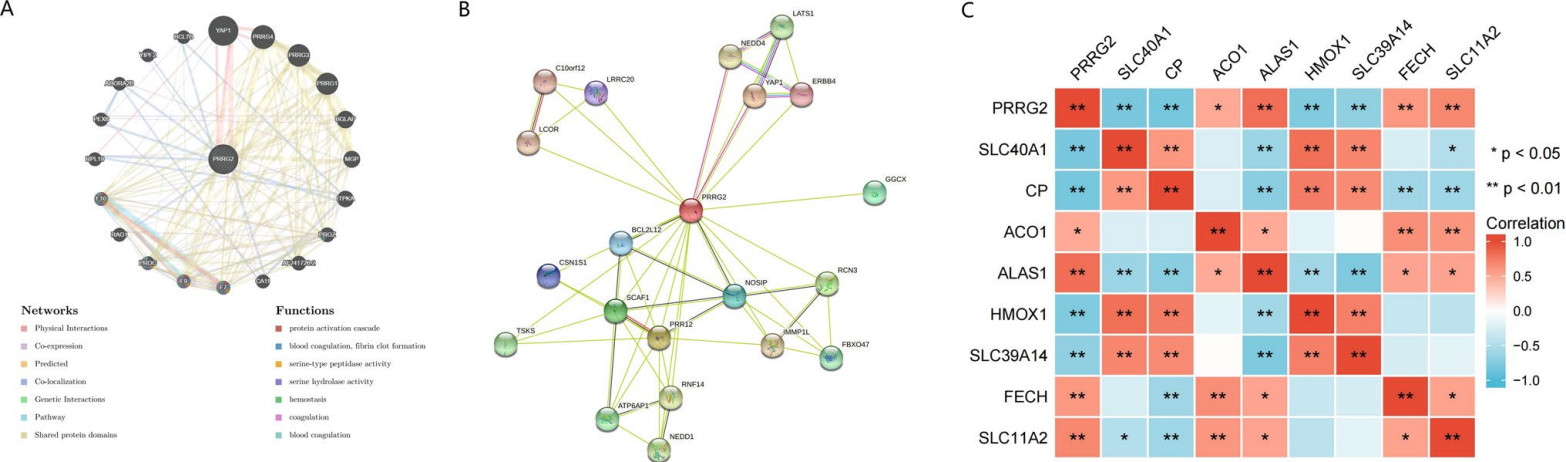
**A** The gene–gene interaction network of PRRG2. **B** The PPI network of PRRG2. **C** A heat map shows the correlations between PRRG2 and related genes in KIRC. *p < 0.05, **p < 0.01

Through the utilization of GeneMania, we have succeeded in constructing a gene–gene interaction network encompassing PRRG2 and its adjacent genes. Our analysis has revealed that the top 20 most frequently mutated genes are intimately linked to PRRG2, with notable examples including YAP1 and PRRG1,3,4 (Fig. 5a). Our functional analysis has further demonstrated that these genes are significantly associated with protein activation cascades and serine-type peptidase activities (Fig. 5a). Furthermore, we have constructed a PRRG2 protein–protein interaction (PPI) network utilizing the STRING database, which comprises of 20 nodes and 64 edges (Fig. 5b). In addition, we have explored the Heatmap of PRRG2-associated genes based on the TCGA database. Our findings indicate a significant positive correlation between PRRG2 and ALAS1, FECH, SLC11A2 in KIRC patients, while revealing a negative correlation with SLC40A1 and CP (Fig. 5c).

### GO and KEGG pathway analysis of PRRG2 and its co-expressed genes in KIRC of TCGA database

**Fig. 6:**
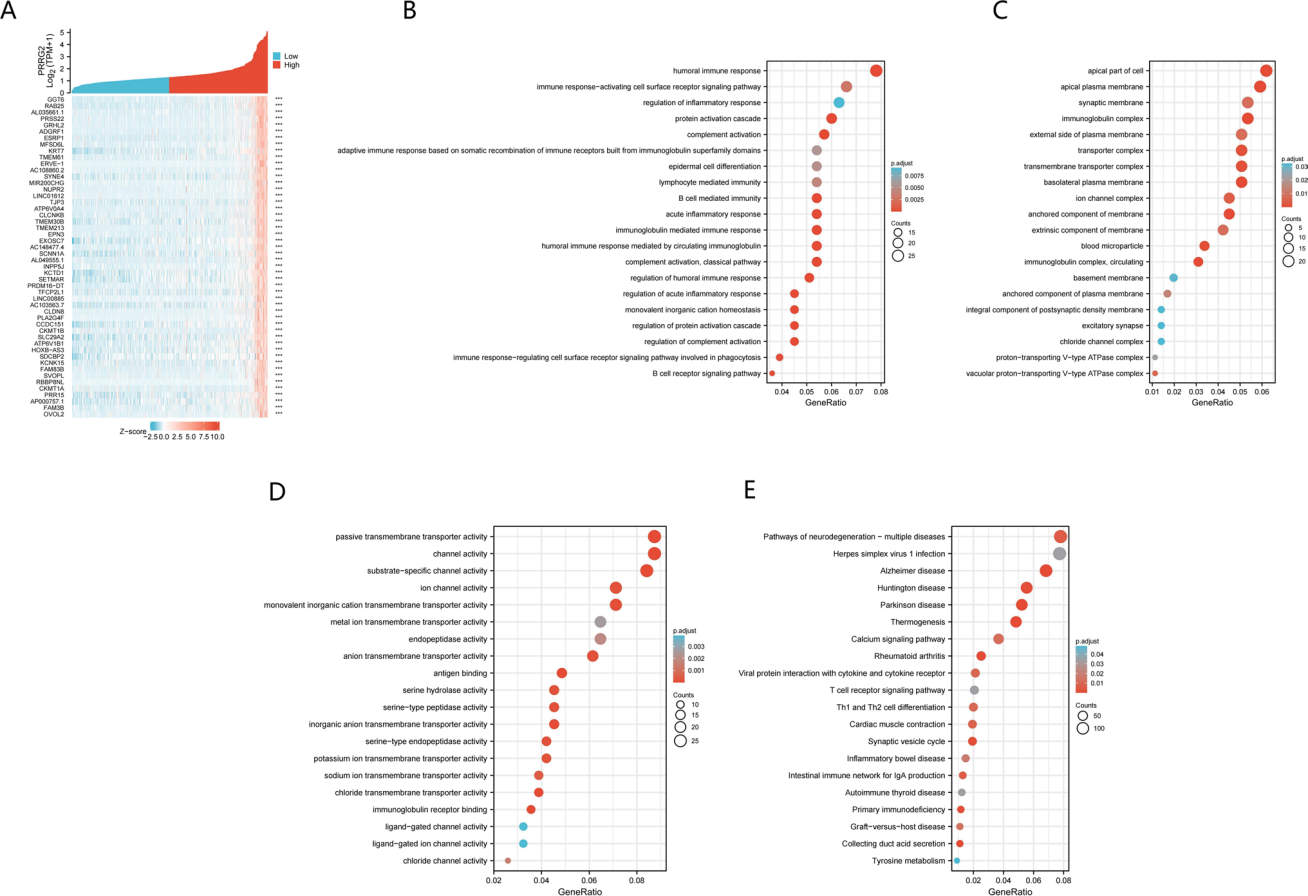
GO and KEGG enrichment analysis for PRRG2. **A** Heat maps showing the top 50 genes positively correlated with PRRG2 in KIRC. **B** Top 20 enrichment terms in BP categories in KIRC. **C** Top 20 enrichment terms in MF categories in KIRC. **D** Top 20 enrichment terms in CC categories in KIRC. **E** Top 20 KEGG enrichment pathways in KIRC

We utilized data mining methods in order to identify genes that exhibited either positive or negative co-expression with PRRG2 within the TCGA database. Through this process, we were able to obtain a heat map of the initial 50 genes within KIRC that demonstrated a positive correlation with PRRG2 (Fig. 6a). Subsequently, we conducted KEGG and GO enrichment analyses on the genes that were positively correlated with PRRG2, with the aim of exploring the relevant pathways and biological functions associated with PRRG2. Ultimately, we identified the top 20 significant items of BP, MF, and CC enrichment analysis, as well as the KEGG pathway (Fig. 6b–e). It is noteworthy that, in terms of BP, PRRG2 exhibited enrichment in a number of immune response-related processes within KIRC, including the human immune response and immune response-activating cell surface receptor signaling pathway, among others.

### GSEA identifies PRRG2 related signaling pathways

**Fig. 7:**
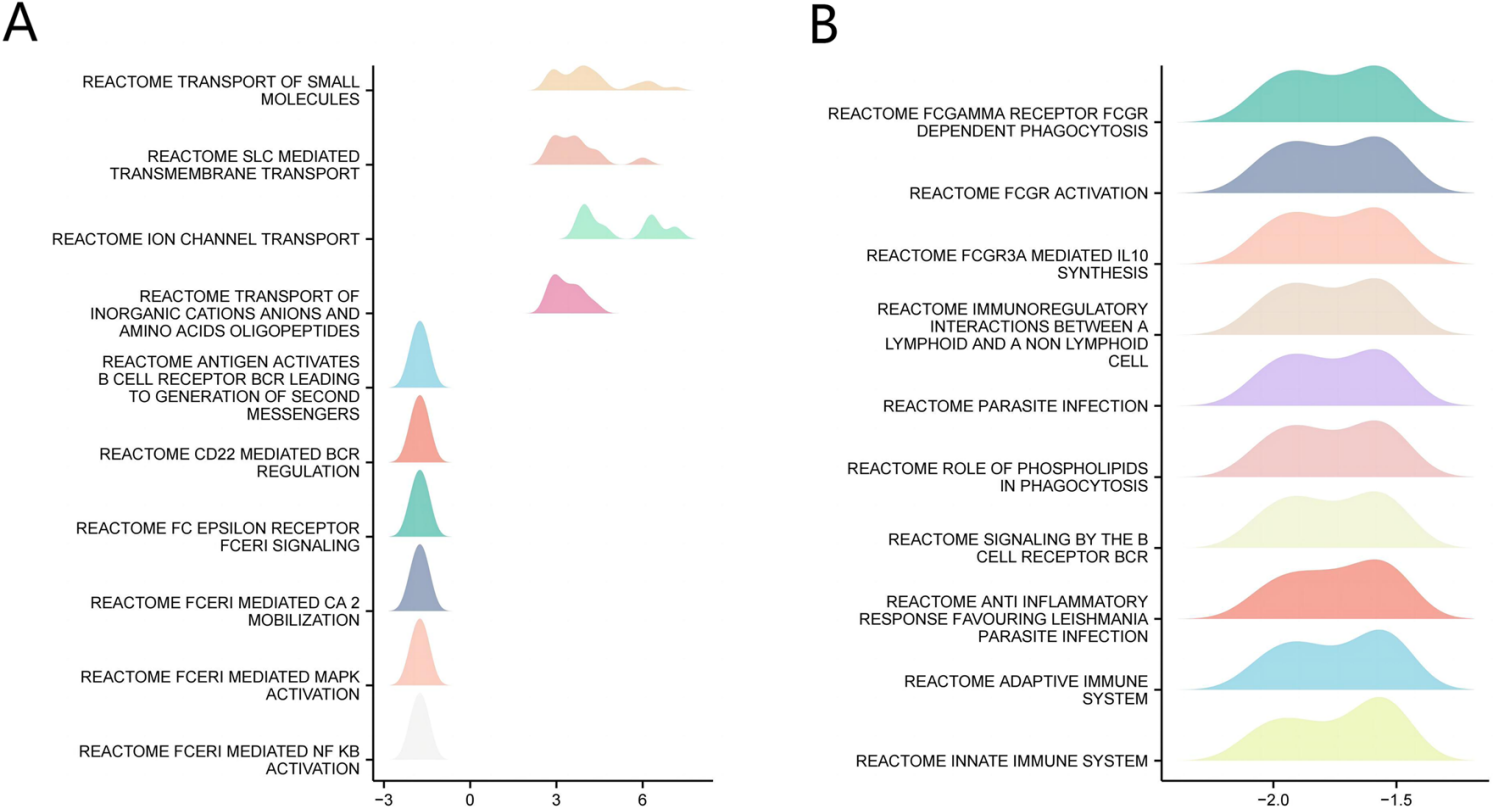
Enrichment plots from GSEA. **A** 1–10 in the top 20. **B** 11–20 in the top 20

The molecular mechanism underlying the influence of PRRG2 on KIRC was further investigated through GSEA enrichment analysis. Among the top 20 KIRC-related signaling pathways of PRRG2, a significant enrichment in immune-related activities were observed in the provided items by the Gene Ontology (GO), including Reactome antigen activities B cell receptor (BCR) leading to the generation of secondary messengers, Reactome CD22-mediated BCR regulation, and Reactome immunoregulatory interactions between a lymphoid and a non-lymphoid cell (Fig. 7a, b). These findings suggest that PRRG2 plays a key role in immune response regulation in renal cancer.

### Analysis of the correlation between the expression of PRRG2 and the infiltration of immune cells

**Fig. 8:**
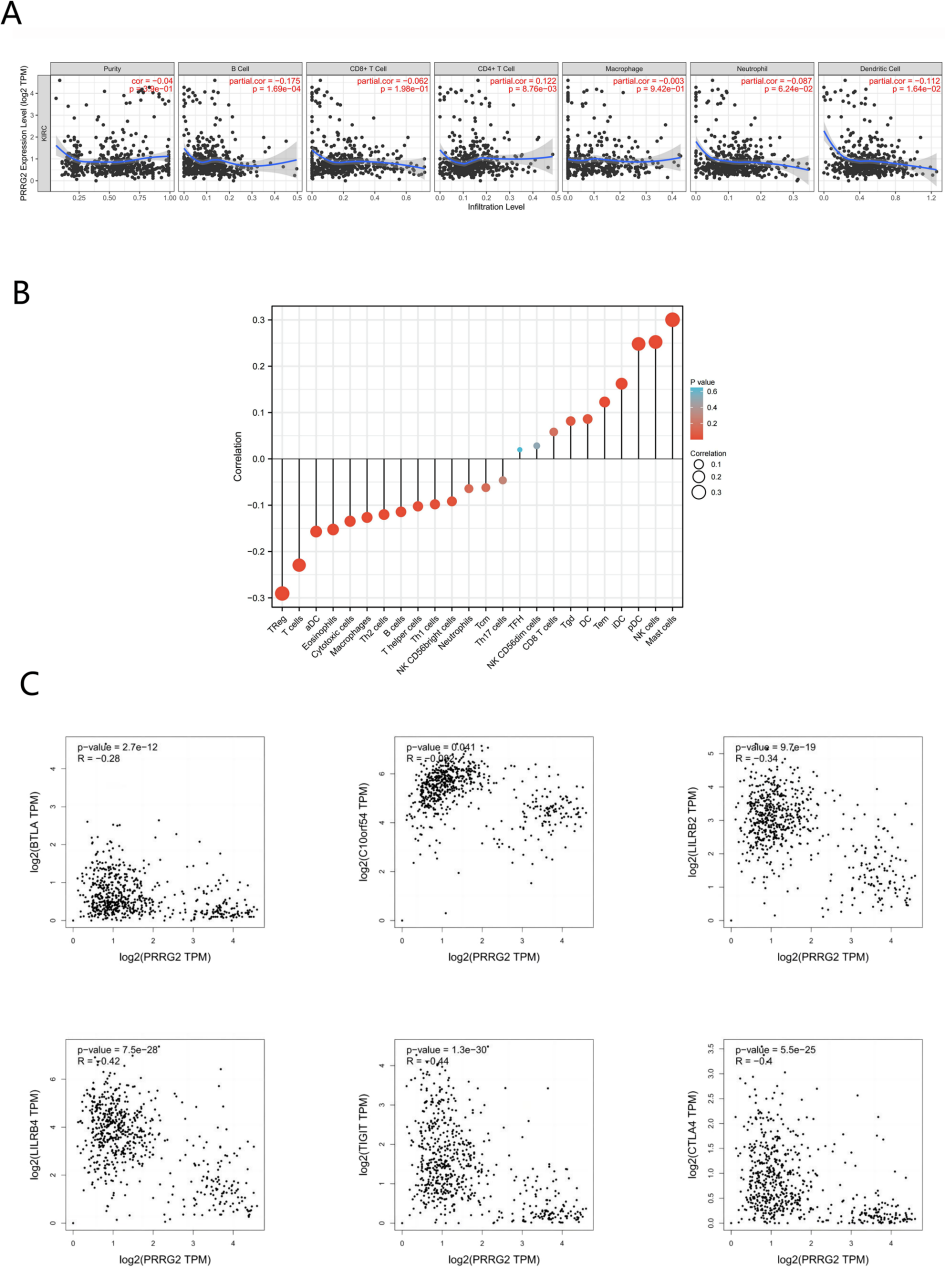
Correlation of PRRG2 expression with immune infiltration level. **A** PRRG2 exhibits a close correlation with tumor purity, while demonstrating varying degrees of correlation with the infiltration levels of various immune cells. **B** PRRG2 expression has a significant correlation with the infiltration of immune cells in KIRC. **C** Scatter plot depicting the correlation between PRRG2 expression and immune cell markers in KIRC

Six types of immune cells, namely B cells, CD8 + T cells, CD4 + T cells, macrophages, neutrophils, and dendritic cells, were analyzed to investigate the correlation between PRRG2 expression and each cell type. The data indicates that the expression levels of PRRG2 in KIRC are correlated to varying degrees with the infiltration of B cells, CD4 + T cells, CD8 + T cells, macrophages, neutrophils, and dendritic cells (Fig. 8a). To gain further insights into the impact of PRRG2 on the tumor microenvironment (TME), we employed CiberSort to examine the relationship between PRRG2 and immune infiltration. The analysis indicated that in KIRC, PRRG2 had a positive correlation with the infiltration levels of Mast cells, NK cells, and plasmoid dendritic cells, while exhibiting a negative correlation with the infiltration levels of regulatory cells (Treg), T cells, eosinophils, cytotoxic cells, and macrophages (Fig. 8b).

### Relationship between PRRG2 expression and different immunological markers

To further substantiate the interplay between PRRG2 and immune responses, we employed the TIMER database to scrutinize the correlation between the expression of PRRG2 in KIRC and diverse immune signals. We meticulously examined the cellular markers comprising B cells, T cells, CD8 + T cells, monocytes, tumor-associated macrophages (TAMs), M1 macrophages, M2 macrophages, neutrophils, NK cells, and dendritic cells (Table 2). In the realm of clinical cancer biopsies, tumor purity is an intrinsic aspect that influences immune infiltration analysis. Following the adjustment for tumor purity, the expression of PRRG2 in several immune cells in KIRC was inextricably linked with an array of immunological markers.

**Table 2:** Correlation analysis between PRRG2 and gene markers of immune cells in TIMER

Description	Gene markers		Cor	*p*
B cell	CD19	Purity	− 0.276023934	< 0.0001
	CD19	PRRG2	− 0.062703714	0.1785
	CD79A	Purity	− 0.322948025	< 0.0001
	CD79A	PRRG2	− 0.120511568	0.0096
T cell (general)	CD3D	Purity	− 0.35241037	< 0.0001
	CD3D	PRRG2	− 0.188923032	< 0.0001
	CD3E	Purity	− 0.346374353	< 0.0001
	CD3E	PRRG2	− 0.209496668	< 0.0001
	CD2	Purity	− 0.358556779	< 0.0001
	CD2	PRRG2	− 0.236388851	< 0.0001
CD8 + T cell	CD8A	Purity	− 0.313969127	< 0.0001
	CD8A	PRRG2	− 0.222984865	< 0.0001
	CD8B	Purity	− 0.305544386	< 0.0001
	CD8B	PRRG2	− 0.180243565	0.0001
Monocyte	CD86	Purity	− 0.316830279	< 0.0001
	CD86	PRRG2	− 0.214051551	< 0.0001
	CSF1R	Purity	− 0.303181403	< 0.0001
	CSF1R	PRRG2	− 0.071944327	0.1225
TAM	CCL2	Purity	− 0.139218287	0.0027
	CCL2	PRRG2	− 0.03091546	0.5074
	CD68	Purity	− 0.136844041	0.0032
	CD68	PRRG2	− 0.235097591	< 0.0001
	IL10	Purity	− 0.29755659	< 0.0001
	IL10	PRRG2	− 0.168183755	0.0003
M1	IRF5	Purity	− 0.052054031	0.2642
	IRF5	PRRG2	− 0.207796041	< 0.0001
	PTGS2	Purity	− 0.160884955	0.0005
	PTGS2	PRRG2	0.053847826	0.2479
	NOS2	Purity	− 0.158691888	0.0006
	NOS2	PRRG2	0.238550206	< 0.0001
M2	CD163	Purity	− 0.223318367	< 0.0001
	CD163	PRRG2	− 0.140793877	0.0024
	VSIG4	Purity	− 0.282802645	< 0.0001
	VSIG4	PRRG2	− 0.1383981	0.0029
	MS4A4A	Purity	− 0.278277548	< 0.0001
	MS4A4A	PRRG2	− 0.157050902	0.0007
Neutrophils	CEACAM8	Purity	0.062860147	0.1774
	CEACAM8	PRRG2	0.025646908	0.5824
	ITGAM	Purity	− 0.247556759	< 0.0001
	ITGAM	PRRG2	− 0.112467692	0.0156
	CCR7	Purity	− 0.303097299	< 0.0001
	CCR7	PRRG2	− 0.105386267	0.0235
Natural killer cell	KIR2DL1	Purity	− 0.092879941	0.0460
	KIR2DL1	PRRG2	0.023991455	0.6070
	KIR2DL3	Purity	− 0.051105756	0.2730
	KIR2DL3	PRRG2	0.0675646	0.1471
	KIR2DL4	Purity	− 0.147715983	0.0015
	KIR2DL4	PRRG2	− 0.113876096	0.0143
	KIR3DL1	Purity	− 0.038165284	0.4131
	KIR3DL1	PRRG2	0.041803679	0.3700
	KIR3DL2	Purity	− 0.072173799	0.1213
	KIR3DL2	PRRG2	0.017008085	0.7154
	KIR3DL3	Purity	− 0.055513828	0.2337
	KIR3DL3	PRRG2	− 0.046573911	0.3178
	KIR2DS4	Purity	− 0.077254534	0.0972
	KIR2DS4	PRRG2	0.036087497	0.4390
Dendritic cell	HLA-DPB1	Purity	− 0.299226666	< 0.0001
	HLA-DPB1	PRRG2	− 0.165760791	0.0004
	HLA-DQB1	Purity	− 0.211614398	< 0.0001
	HLA-DQB1	PRRG2	− 0.106505338	0.0220
	HLA-DRA	Purity	− 0.31065338	< 0.0001
	HLA-DRA	PRRG2	− 0.228682179	< 0.0001
	HLA-DPA1	Purity	− 0.321254581	< 0.0001
	HLA-DPA1	PRRG2	− 0.228203935	< 0.0001
	CD1C	Purity	− 0.238559928	< 0.0001
	CD1C	PRRG2	0.111045408	0.0170
	NRP1	Purity	− 0.130216686	0.0051
	NRP1	PRRG2	0.140816147	0.0024
	ITGAX	Purity	− 0.115600257	0.0129
	ITGAX	PRRG2	− 0.187225325	0.0001

We also investigated the association between PRRG2 expression and diverse functional T cells, encompassing Th1, Th1-like, Th2, Treg, quiescent Tregs, activated Tregs, effector T cells, naïve T cells, effector memory T cells, resistant memory T cells, and exhausted T cells. Through analysis of the TIMER database, it was observed that the expression levels of PRRG2 were notably correlated with 22 out of 38 T cell markers in KIRC (Table 3).

**Table 3:** Correlation analysis between PRRG2 and gene markers of different types of T cells in TIMER

Description	Gene markers		Cor	*p*
Th1	TBX21	Purity	− 0.210921179	< 0.0001
	TBX21	PRRG2	0.003784276	0.9353
	STAT4	Purity	− 0.268720921	< 0.0001
	STAT4	PRRG2	− 0.183397976	0.0001
	STAT1	Purity	− 0.274215279	< 0.0001
	STAT1	PRRG2	− 0.193243903	< 0.0001
	TNF	Purity	− 0.176012779	0.0001
	TNF	PRRG2	− 0.044906	0.3355
	IFNG	Purity	− 0.320725367	< 0.0001
	IFNG	PRRG2	− 0.216310625	< 0.0001
Th1	HAVCR2	Purity	− 0.137984485	< 0.0001
	HAVCR2	PRRG2	− 0.218764023	< 0.0001
	CXCR3	Purity	− 0.340811169	< 0.0001
	CXCR3	PRRG2	− 0.185923058	0.0001
	BHLHE40	Purity	0.005451676	0.9070
	BHLHE40	PRRG2	0.073507507	0.1146
	CD4	Purity	− 0.32088774	< 0.0001
	CD4	PRRG2	− 0.127573766	0.0061
Th2	STAT6	Purity	0.063274567	0.1746
	STAT6	PRRG2	0.06273064	0.1783
	STAT5A	Purity	− 0.325177185	0.0000
	STAT5A	PRRG2	− 0.025807492	0.5799
Treg	FOXP3	Purity	− 0.302287131	< 0.0001
	FOXP3	PRRG2	− 0.229912236	< 0.0001
	CCR8	Purity	− 0.31846569	< 0.0001
	CCR8	PRRG2	− 0.230776977	< 0.0001
	TGFB1	Purity	− 0.180963867	0.0001
	TGFB1	PRRG2	0.081097721	0.0816
	IL2RA	Purity	− 0.242180119	< 0.0001
	IL2RA	PRRG2	− 0.13465945	0.0038
	TNFRSF9	Purity	− 0.273408526	< 0.0001
	TNFRSF9	PRRG2	− 0.249711623	< 0.0001
Effector T-cell	CX3CR1	Purity	− 0.171689903	0.0002
	CX3CR1	PRRG2	− 0.00866456	0.8526
	FGFBP2	Purity	− 0.054246617	0.2446
	FGFBP2	PRRG2	0.190327221	< 0.0001
	FCGR3A	Purity	− 0.296693834	< 0.0001
	FCGR3A	PRRG2	− 0.246587991	< 0.0001
Naïve T-cell	CCR7	Purity	− 0.303097299	< 0.0001
	CCR7	PRRG2	− 0.105386267	0.0013
	SELL	Purity	− 0.389449973	< 0.0001
	SELL	PRRG2	− 0.146966759	< 0.0001
Effector memory T-cell	DUSP4	Purity	− 0.329301345	< 0.0001
	DUSP4	PRRG2	0.000410645	0.9930
	GZMK	Purity	− 0.328798105	< 0.0001
	GZMK	PRRG2	− 0.218188615	< 0.0001
	GZMA	Purity	− 0.322096222	0.0000
	GZMA	PRRG2	− 0.207935012	< 0.0001
Resident memory T-cell	CD69	Purity	− 0.32130781	< 0.0001
	CD69	PRRG2	− 0.182952527	0.0001
	CXCR6	Purity	− 0.327048999	< 0.0001
	CXCR6	PRRG2	− 0.229833982	< 0.0001
	MYADM	Purity	− 0.056553247	0.2250
	MYADM	PRRG2	0.07612781	0.1022
General memory T-cell	IL7R	Purity	− 0.265680935	< 0.0001
	IL7R	PRRG2	− 0.081240035	0.0811
	CCR7	Purity	− 0.303097299	< 0.0001
	CCR7	PRRG2	− 0.105386267	0.0013
Exhausted T-cell	LAG3	Purity	− 0.282267711	< 0.0001
	LAG3	PRRG2	− 0.228424141	< 0.0001
	CXCL13	Purity	− 0.277209631	< 0.0001
	CXCL13	PRRG2	− 0.252372541	< 0.0001
	LAYN	Purity	− 0.070229144	0.1317
	LAYN	PRRG2	0.210781479	< 0.0001

We have further investigated the correlation between PRRG2 expression and immune cells such as Treg and NK cells' checkpoint molecules, including BTLA, VISTA, LILRB2, LILRB4, TIGIT, and CTLA-4, using the GEPIA database (Fig. 8c). These findings further support the significant correlation between PRRG2 expression and immune infiltration, suggesting a pivotal role of PRRG2 in immune escape within the microenvironment of KIRC.

### The correlation between PRRG2 expression in immune cells and prognosis in patients with KIRC

**Fig. 9:**
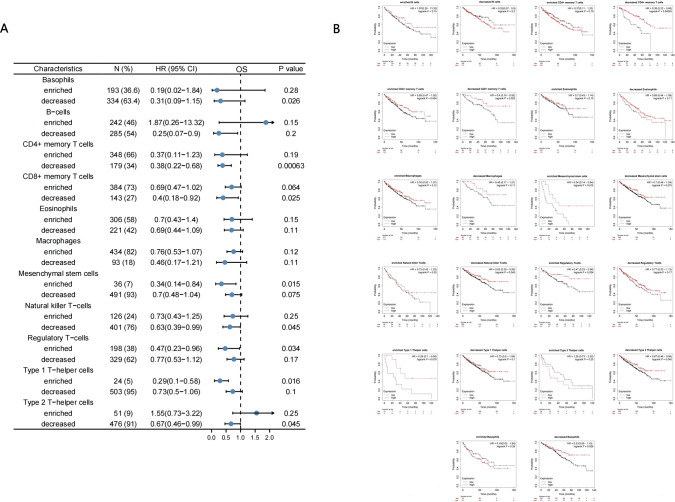
Kaplan–Meier survival curves according to high and low expression of PRRG2 in immune cell subgroups in KIRC. **A** A forest plot shows the prognostic value of PRRG2 expression according to different immune cell subgroups in KIRC patients. **B** Correlations between PRRG2 expression and OS in different immune cell subgroups in KIRC patients were estimated by Kaplan–Meier plotter

Given the close association between low expression of PRRG2 and immune invasion and poor prognosis in KIRC, we investigated whether the expression of PRRG2 is influenced by immune invasion and thus affects the prognosis of KIRC. We conducted a prognostic analysis based on the expression level of PRRG2 in the relevant immune cell subgroups in KIRC. As depicted in Fig. 9a, b, patients with KIRC who exhibit low expression of PRRG2 experience a poorer prognosis due to decreased infiltration of CD4 + and CD8 + memory T cells, NK cells, Type 2 T − helper cells (Th2) and eosinophils, as well as enriched infiltration of regulatory T cells, Mesenchymal stem cells and Type 1 T − helper cells (Th1). These findings indicate that PRRG2 may impact the prognosis of KIRC patients, in part due to immune infiltration.

## Discussion

KIRC, a predominant neoplasm within the urinary framework, was diagnosed in 66,800 new instances across China in 2015, constituting 1.56% of all freshly identified malignancies, thereby securing the 14th position in prevalence. This affliction resulted in 23,400 fatalities, equating to 0.83% of all oncological mortalities, and held the 17th rank in this grim tally [16]. Despite surgical excision being the primary therapeutic intervention for RCC, up to 40% of patients experience local recurrence and distant metastasis [17]. To augment patient longevity, the discernment of early diagnostic and prognostic biomarkers is of utmost significance. Presently, inquiries pertaining to PRRG2 are scant, with no clinical trials explicitly scrutinizing its role in malignancies. Our revelations denote a substantial correlation between PRRG2 expression and diverse clinical pathological indicators in renal cell carcinoma, alongside a significant association with OS and DFS, particularly in relation to tumor-infiltrating immune cells. The integration of PRRG2 with some universally acknowledged KIRC prognostic biomarkers, such as VHL, PBRM1, and BAP1, may furnish a more precise prognostic prediction for KIRC [18–20]. Our results suggest that PRRG2 may serve as a potential prognostic biomarker and therapeutic target in KIRC.

In this study, we have demonstrated through bioinformatics analysis using Timer, Oncomine, UALCAN, and TCGA public databases that the expression of PRRG2 is lower in KIRC tissue compared to normal renal tissue. Subsequent to our qPCR and immunohistochemistry validation, we discerned a marked downregulation of PRRG2 at both transcriptional and translational levels within cancerous tissue, in contrast to its adjacent normal tissue. Moreover, when juxtaposed with normal renal epithelial cells HK-2, PRRG2 expression exhibits a similar downregulation in the majority of kidney cancer cell lines. In addition, immune cells and stromal cells in TME can promote or inhibit tumor growth, and the occurrence and development of tumors are closely related to the interaction between tumor cells and TME [21, 22].

From the PPI network of PRRG2 and its co-expression gene map in PRRG2-related differential genes, it is evident that the expression of PRRG2 is closely associated with numerous proteins, particularly YAP1. YAP1 (Yes-associated protein 1) is a critical signaling molecule that plays a significant role in various biological processes such as cell proliferation, apoptosis, differentiation, stem cell self-renewal, and organ size [23–25]. Research has shown that YAP1 plays a significant role in the onset and progression of tumors, with high expression of YAP1 closely associated with the occurrence and progression of various cancers, including esophageal cancer, pancreatic cancer, lung cancer, thyroid cancer, and gastric cancer [23, 26–28]. The elevated expression of YAP1 has been shown to facilitate tumor cell proliferation, suppress apoptosis, promote cell migration and invasion, and enhance self-renewal of stem cells. For instance, YAP1 can interact with AMOTL1 (Angiomotin-like protein 1) to modulate YAP1's transcriptional activity and nuclear localization. Specifically, AMOTL1 can inhibit YAP1's nuclear translocation and transcriptional activity, thereby suppressing cell proliferation and promoting apoptosis. Additionally, AMOTL1 can influence cell differentiation and tissue development by regulating downstream gene expression of YAP1, such as CTGF, CYR61, and ANKRD1 [28, 29]. Therefore, the interaction and regulatory relationship between YAP1 and AMOTL1 are of significant importance for comprehending the mechanisms of cellular biology and tumorigenesis. Additionally, YAP1 can participate in the occurrence and progression of tumors by regulating various signaling pathways, such as MAPK, AKT, and Hippo [30–32]. Under normal circumstances, the activity of YAP1 is suppressed by the Hippo signaling pathway. However, in tumor cells, the Hippo signaling pathway is often inactivated, leading to excessive activation of YAP1 [33, 34]. Consequently, we posit that PRRG2 could potentially impede tumor proliferation and metastasis through the activation of the Hippo signaling cascade, thereby inhibiting YAP1 activity.

More importantly, we have observed a correlation between the expression of PRRG2 in KIRC and various immune cell infiltrations, with the most prominent being Treg and NK cells. Treg is a specialized subset of T cells that primarily function to regulate immune response by suppressing the activity of other immune cells. In tumors, Treg can promote tumor growth and metastasis by inhibiting the activity of other immune cells. Our research has found a significant negative correlation between Treg infiltration and PRRG2, as well as between PRRG2 and BTLA, VISTA, TIGIT, and CTLA4, which are closely related to Treg. BTLA, or B and T Lymphocyte Attenuator, is an immune inhibitory molecule that can suppress the activation and function of T cells by binding to its ligand HVEM, or Herpes Virus Entry Mediator [35, 36]. BTLA can enhance the immunosuppressive capacity of Treg by binding to HVEM on the surface of Treg, thereby suppressing the attack of other immune cells on tumor cells and resulting in immune escape [37]. VISTA (V-domain Ig-containing Suppressor of T cell Activation) is also a key factor in tumor immune escape. VISTA is a negative immune regulatory receptor that can inhibit the activity of T cells, thereby suppressing tumor immune response [38]. Treg cells also have the ability to express TIGIT (T cell immunoglobulin and immunoreceptor tyrosine-based inhibitory motif domain family members). Through binding with ligands on the surface of other immune cells, they can suppress the activity of these cells [39]. In the context of cancer, TIGIT can inhibit the attack of other immune cells on tumor cells, thereby promoting tumor growth and metastasis, and has become an important target in cancer treatment [40, 41]. CTLA4, a T-cell co-stimulatory molecule, primarily functions by binding with co-stimulatory molecules CD80/CD86, thereby inhibiting T-cell activity and weakening immune response [42], ultimately promoting tumor growth and metastasis [43]. Furthermore, we have observed a significant negative correlation between LILRB2, LILRB4 and PRRG2, which are closely associated with NK cells. The relationship between LILRB and NK cells mainly involves the regulatory function of LILRB on NK cells. LILRB is a type of receptor associated with immune regulation, whose main function is to inhibit the activity of immune cells by binding to its ligand [44]. In NK cells, after LILRB binds to its ligand, it can inhibit the killing activity and cytokine secretion ability of NK cells. Besides regulating the activity of NK cells, LILRB can also affect the development and differentiation of NK cells [45, 46]. Various immunotherapeutic drugs have been developed for these immune cells and biomarkers, such as anti-CTLA4 monoclonal antibodies and compounds that inhibit Treg function, for the treatment of tumors. In recent times, numerous nanocarrier systems, including nanoparticles, liposomes, micelles, and polymers, have been developed for selective delivery of various anticancer molecules and drugs to tumor sites. Nanocarrier systems have potential advantages such as improved drug solubility, prolonged circulation, controlled release, and targeted delivery [47].

Crucially, PRRG2 exhibits significant correlations with a spectrum of immune cell markers in KIRC, which in turn modulate tumor cell dynamics and the TME [22], thereby impacting the survival duration of KIRC patients. This further confirms and expands upon our previous perspectives on immune infiltration. Notably, a significant reduction in survival time is observed in the PRRG2 low-expression group when CD4 + memory T cells decrease. Moreover, in tumor therapy, enhancing tumor immune response, suppressing tumor growth and metastasis, and improving therapeutic efficacy can be effectively achieved by regulating the balance of Th1/Th2 cells [48, 49]. For instance, the use of immune modulators, cytokines, and tumor vaccines can promote the production and activity of Th1 cells while inhibiting the production and activity of Th2 cells, thus enhancing tumor immune response and improving therapeutic efficacy [49, 50]. These findings suggest that PRRG2 may be a novel immunotherapeutic target for KIRC. However, the exact role of PRRG2 in the tumor immune microenvironment requires further exploration.

This study has enhanced our understanding of the relationship between PRRG2 and KIRC, but there are still some limitations. Firstly, although we have investigated the correlation between PRRG2 and immune infiltration in KIRC patients, the molecular mechanisms and roles of PRRG2 in tumor growth, metastasis, and immune evasion require further study. Secondly, most of the analyses were based on the mRNA levels of PRRG2 in this study. A deeper analysis based on protein levels would make the data more convincing. Thirdly, we did not investigate the diagnostic and prognostic value of PRRG2 in other renal cancers in this study.

## Conclusion

PRRG2 has the potential to serve as a novel prognostic biomarker for KIRC. Additionally, there is potential evidence that PRRG2 regulates immune cell infiltration in the TME of KIRC patients. Therefore, these findings hold potential value in enhancing our current understanding of PRRG2 and its applications in KIRC prognosis and immunotherapy.

### Supplementary Information

**Additional file 1.**

## Data Availability

The datasets used or analyzed during the current study are available from the corresponding authors on reasonable request.
