# Supplementary figures and images for: Low expression of PRRG2 in kidney renal clear cell carcinoma: an immune infiltration-associated prognostic biomarker
Source: Discov Oncol. 2024 Jan 16;15:9. doi: 10.1007/s12672-024-00864-x (PMC10792142; doi:10.1007/s12672-024-00864-x)

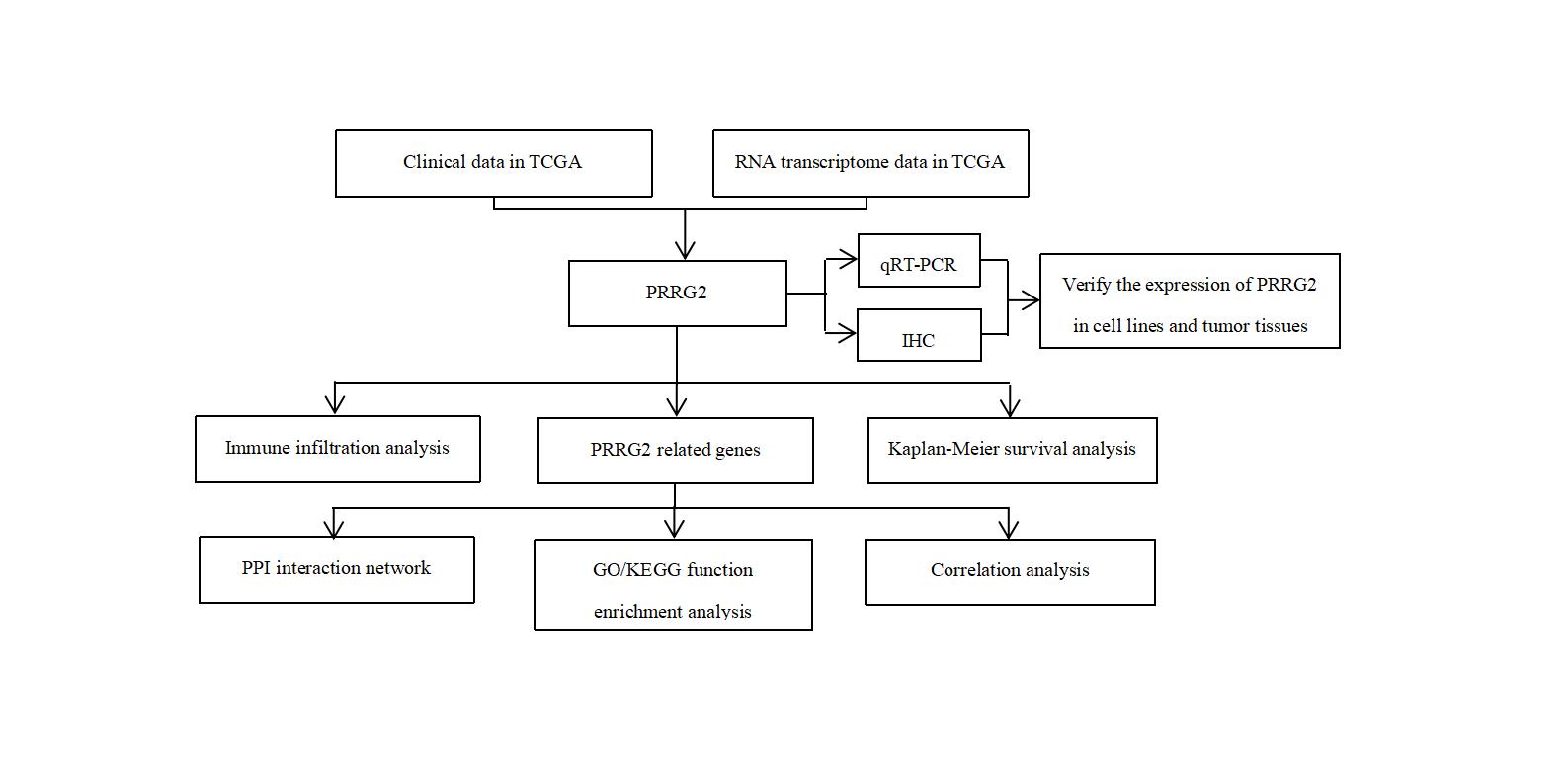

Supplement: Supplementary file 1 — Additional file 1. [file 12672_2024_864_MOESM1_ESM.jpg]
